# Association of Serum Uric Acid Level as a Risk Factor and Severity Marker With Acute Ischemic Stroke

**DOI:** 10.7759/cureus.103179

**Published:** 2026-02-07

**Authors:** Ganesh K Dad, Vijaykumar G Warad, Shravankumar Potkar, Shridhar Patil

**Affiliations:** 1 General Medicine, Shri B.M. Patil Medical College Hospital and Research Centre, Bijapur Lingayat District Educational Association (BLDE) (Deemed University), Vijayapura, IND

**Keywords:** acute ischemic stroke, hyperuricemia, modified rankin scale, serum uric acid, stroke severity

## Abstract

Introduction

Stroke is a major global health burden and a leading cause of mortality. Serum uric acid (SUA) exhibits a dual biological role, acting as an antioxidant at physiological levels while contributing to oxidative stress and endothelial dysfunction when elevated. The association between SUA levels and stroke severity remains controversial. The study aimed to evaluate the association between serum UA levels and acute ischemic stroke and to assess its relationship with functional disability as measured by the Modified Rankin Scale (mRS).

Materials and methods

This observational cross-sectional study was conducted from June 2024 to December 2025. A total of 63 adult patients with radiologically confirmed acute ischemic stroke presenting within 48 hours of symptom onset were included. SUA levels were measured using the uricase method. Stroke severity was assessed using the mRS at admission. Data were analyzed using SPSS version 26. Comparison across mRS categories was performed using one-way analysis of variance (ANOVA), and correlation was assessed using Spearman’s rank correlation coefficient.

Results

The mean age of participants was 59.4±14.5 years, with male predominance (40; 63.5%). Hypertension (23; 36.5%) and diabetes mellitus (16; 25.4%) were the most common comorbidities. The mean SUA level was 8.0±1.5 mg/dL. Most patients had moderate disability, with 40 patients (63.5%) having an mRS score of 3 and 22 (34.9%) an mRS score of 4. Mean SUA levels increased progressively with stroke severity (p<0.0001). A strong correlation was observed between SUA levels and mRS scores (ρ=0.89, p<0.001).

Conclusion

Elevated SUA levels are associated with greater functional disability in patients with acute ischemic stroke at presentation. These findings indicate an associative relationship between SUA levels and stroke-related disability rather than a causal or independent prognostic role. Larger prospective studies are required to further clarify the clinical significance of this association.

## Introduction

Stroke is defined by the World Health Organization as the rapid onset of focal or global neurological deficits lasting more than 24 hours or leading to death, with no apparent cause other than a vascular origin [1]. It remains a major public health concern worldwide and is currently the second leading cause of mortality after coronary artery disease [2,3]. The lifetime risk of stroke is substantial, with estimates suggesting that one in five women and one in six men will experience a stroke after the age of 55 years [4]. In India, stroke represents the third leading cause of death, accounting for a significant proportion of disability-adjusted life years (DALYs) lost [5].

Acute ischemic stroke, the most common subtype of stroke, results from cerebral arterial occlusion leading to reduced blood flow, energy failure, and neuronal injury [6]. Oxidative stress and inflammatory mechanisms play a pivotal role in the pathophysiology of ischemic brain injury [7]. Uric acid (UA), the final product of purine metabolism in humans, is excreted mainly by the kidneys to maintain physiological balance [8]. UA contributes nearly half of the total antioxidant capacity of human plasma and acts as an important scavenger of reactive oxygen species (ROS), suggesting a potential neuroprotective role during cerebral ischemia [9].

Despite its antioxidant properties, elevated serum uric acid (SUA) levels have been consistently associated with hypertension, abdominal obesity, metabolic syndrome, dyslipidemia, and elevated triglyceride and cholesterol levels [10]. At higher concentrations, uric acid may exert pro-oxidant effects, promoting endothelial dysfunction, vascular inflammation, platelet activation, and impaired nitric oxide bioavailability, thereby contributing to atherosclerosis and increased susceptibility to ischemic stroke [11,12]. This dual and paradoxical role of uric acid, protective at physiological levels and potentially harmful when elevated, has generated considerable interest in its role in cerebrovascular disease [9].

Given the high burden of ischemic stroke and the widespread availability of SUA estimation, clarifying its association with stroke severity has important clinical implications. Therefore, the present study aimed to evaluate the association between SUA levels and functional disability in patients with acute ischemic stroke as measured by the modified Rankin Scale at admission.

## Materials and methods

Study design

This hospital-based observational cross-sectional study was conducted at BLDE (Deemed to be University), Shri B.M. Patil Medical College Hospital and Research Centre, Vijayapura, Karnataka, from March 2024 to December 2024. The study included patients admitted to the Department of General Medicine with a diagnosis of acute ischemic stroke during the study period.

Ethical considerations

The study protocol was reviewed and approved by the Institutional Ethics Committee of BLDE (Deemed to be University) (IEC No.: BLDE (DU)/IEC-SBMPMC/041/2023-24). Written informed consent was obtained from all participants or their legally authorized representatives prior to enrollment. The study was conducted in accordance with the ethical principles outlined in the Declaration of Helsinki.

Inclusion and exclusion criteria

Patients aged ≥18 years with radiologically confirmed acute ischemic stroke (computed tomography or magnetic resonance imaging) presenting within 48 hours of symptom onset were included.

Patients were excluded if they had stroke secondary to aneurysm, neoplasm, coagulation disorders, trauma, subarachnoid hemorrhage, intraventricular hemorrhage, or malignancy. Additionally, patients with a history of gouty arthritis, acute or chronic kidney disease, or those receiving medications known to alter SUA levels, such as uricosuric agents or xanthine oxidase inhibitors, were excluded. Patients with known conditions or interventions that could markedly influence uric acid metabolism, such as recent bariatric surgery or active weight-loss therapy, were also excluded where documented.

Sample size calculation

The sample size was calculated based on mean SUA levels as reported by Mohammad Ibrahim Khalil et al. [13], 6.03±1.84 mg/dL. Using the formula for estimation of a mean:

$n = \left( \frac{Z \sigma}{d} \right)^2$

where *Z*=1.96 (95% confidence level), σ=1.84 mg/dL, and d=0.5 mg/dL (allowable error). The minimum required sample size was calculated to be 52, which was increased to 63 participants to account for possible attrition and incomplete data.

Data collection and biochemical analysis

A detailed clinical history and thorough general and systemic examination were performed for all participants. For biochemical assessment, 2.5 mL of venous blood was collected in a plain vacutainer in the random (non-fasting) state at the time of admission, prior to initiation of thrombolysis or other acute therapeutic interventions. SUA levels were measured using the uricase method on a Vitros 5.1 autoanalyzer (Ortho Clinical Diagnostics, Rochester, NY, USA) with a reference range of 2.6-8.2 mg/dL considered normal [14].

Assessment of stroke severity (modified Rankin scale)

Stroke severity and functional outcome were assessed using the modified Rankin Scale (mRS) at the time of admission. The mRS is a validated and widely accepted clinician-reported outcome measure used to assess global disability following stroke.

The scale ranges from 0 to 6, where 0=No symptoms; 1=No significant disability despite symptoms; 2=Slight disability; 3=Moderate disability requiring some help but able to walk unassisted; 4=Moderately severe disability requiring assistance for walking and bodily needs; 5=Severe disability, bedridden, requiring constant care; 6=Death.

Higher mRS scores indicate greater functional impairment.

mRS is an open-access tool and does not require permission for use. The original scale was developed by Rankin (1957) and later modified and validated by van Swieten et al. (1988) [15,16].

Statistical analysis

Data were entered into Microsoft Excel (Microsoft, Redmond, WA) and analyzed using SPSS version 26 (IBM Corp, Armonk, NY). Continuous variables were expressed as mean±standard deviation, while categorical variables were summarized as frequencies and percentages. Comparison of mean SUA levels across mRS categories was performed using one-way analysis of variance (ANOVA) and interpreted cautiously due to unequal group sizes. Correlation was assessed using Spearman’s rank correlation coefficient. A p-value <0.05 was considered statistically significant.

## Results

The study population had a mean age of 59.4±14.5 years (range: 30-85 years) and demonstrated a male predominance, with men accounting for 40 patients (63.5%) and women for 23 patients (36.5%). Among the associated comorbidities, hypertension was the most common, present in 23 (36.5%) patients, followed by diabetes mellitus in 16 (25.4%), while dyslipidemia and coronary heart disease were observed in seven (11.1%) patients each (Table 1).

**Table 1:** Baseline characteristics A summary the baseline demographic profile and associated comorbidities of the study participants is presented. Continuous variables are expressed as mean±standard deviation, and categorical variables are presented as frequency and percentage. Statistical analysis: Descriptive statistics were used; no inferential statistical test was applied. Abbreviations: SD: Standard deviation

Variable		Value
Age (years)	Mean±SD	59.4±14.5
Gender	Male	40 (63.5)
	Female	23 (36.5)
Comorbidities	Diabetes mellitus	16 (25.4)
	Hypertension	23 (36.5)
	Dyslipidemia	7 (11.1)
	Coronary heart disease	7 (11.1)

The mean pulse rate of the participants was 84.1±11.8 bpm. Vitals indicated that a considerable proportion of patients had elevated blood pressure values at presentation (Table 2).

**Table 2:** Vital parameters of study participants (n=63) Baseline vital parameters of the study population at admission, expressed as mean±standard deviation, are presented. Statistical analysis: Descriptive statistics were used; no inferential statistical test was applied. Abbreviations: SD: Standard deviation; bpm: beats per minute; mmHg: millimeters of mercury.

Parameter	Mean±SD
Pulse rate (beats/min)	84.1±11.8
Systolic blood pressure (mmHg)	135.3±13.0
Diastolic blood pressure (mmHg)	89.7±9.2

Laboratory evaluation showed a mean blood urea level of 31.4±22.3 mg/dL and a mean serum creatinine of 1.1±0.6 mg/dL. The mean SUA concentration was 8.0±1.5 mg/dL. Electrolyte parameters, including sodium, potassium, chloride, calcium, and phosphorus, were largely within normal physiological ranges. Hematological and metabolic parameters revealed a mean hemoglobin of 12.6±2.3 g/dL, a total leukocyte count of 8115±4464 /mm³, and lipid profile values showing mean low-density lipoprotein (LDL), high-density lipoprotein (HDL), and very low-density lipoprotein (VLDL) levels of 79.6±37.4 mg/dL, 45.9±17.7 mg/dL, and 35.1±14.8 mg/dL, respectively, with a mean random blood sugar level of 111.0±13.0 mg/dL (Table 3).

**Table 3:** Laboratory parameters of study participants (n = 63) The laboratory, biochemical, hematological, and metabolic parameters of the study participants, expressed as mean±standard deviation, are presented. Statistical analysis: Descriptive statistics were used; no inferential statistical test was applied. Abbreviations: SD: Standard deviation; SUA: serum uric acid; LDL: low-density lipoprotein; HDL: high-density lipoprotein; VLDL: very low-density lipoprotein; mg/dL: milligrams per deciliter; mEq/L: milliequivalents per liter; /mm³: per cubic millimeter.

Parameter	Mean ± SD	Normal value (reference range)
Blood urea (mg/dL)	31.4±22.3	15-40 mg/dL
Serum creatinine (mg/dL)	1.1±0.6	0.6-1.2 mg/dL
Serum uric acid (mg/dL)	6.0±1.0	3-7 mg/dL
Serum calcium (mg/dL)	9.5±1.0	8.5-10.5 mg/dL
Serum phosphorus (mg/dL)	4.4±0.9	2.5-4.5 mg/dL
Serum sodium (mEq/L)	136.9±5.6	135-145 mEq/L
Serum potassium (mEq/L)	4.4±0.6	3.5-5.0 mEq/L
Serum chloride (mEq/L)	102.2 ± 4.5	98-106 mEq/L
Hemoglobin (g/dL)	12.6±2.3	12-16 g/dL (female) / 13 – 17 g/dL (male)
Total leukocyte count (/mm³)	8115±4464	4,000-11,000 /mm³
LDL cholesterol (mg/dL)	79.6±37.4	<100 mg/dL
HDL cholesterol (mg/dL)	45.9±17.7	>40 mg/dL (male) / > 50 mg/dL (female)
VLDL cholesterol (mg/dL)	35.1±14.8	5-40 mg/dL
Random blood sugar (mg/dL)	111.0±13.0	<140 mg/dL

Assessment of stroke severity using the MRS revealed that the majority of patients had moderate functional disability at presentation, with 40 (63.5%) patients having an mRS score of 3 and 22 (34.9%) having a score of 4, while only one (1.6%) patient had severe disability (mRS score=5). A progressive increase in mean SUA levels was observed with increasing mRS. The difference across mRS categories was statistically significant on one-way ANOVA (p<0.0001), although interpretation was cautious due to unequal group sizes (Table 4).

**Table 4:** Distribution of stroke severity and corresponding serum uric acid levels (n=63) The distribution of stroke severity based on Modified Rankin Scale scores and the corresponding mean serum uric acid levels is shown. A progressive increase in mean serum uric acid levels was observed with increasing mRS scores. The difference across mRS categories was statistically significant on one-way ANOVA (p<0.0001), although interpretation was cautious due to unequal group sizes. Abbreviations: mRS: Modified Rankin Scale; SUA: serum uric acid; SD: standard deviation.

Modified Rankin Scale (mRS) score	Number of patients, n (%)	SUA (mg/dL), Mean ± SD
3	40 (63.5)	6.5±1.3
4	22 (34.9)	7.0±1.7
5	1 (1.6)	7.6
F-value	20.658	
p-value	<0.0001	

Correlation analysis demonstrated a strong positive association between SUA levels and stroke severity (Table 5).

**Table 5:** Correlation between SUA levels and stroke severity The correlation between serum uric acid levels and stroke severity assessed by the Modified Rankin Scale is shown. Statistical analysis: Correlation was assessed using Spearman’s rank correlation coefficient. Abbreviations: SUA: serum uric acid; mRS: Modified Rankin Scale.

Variables	Spearman’s rank correlation coefficient	p-value
SUA vs mRS	0.89	< 0.001

## Discussion

Uric acid exhibits a complex biological behavior, acting as a free radical scavenger under physiological conditions while promoting oxidative stress, endothelial dysfunction, and vascular inflammation when present in elevated concentrations. This dual role has generated considerable interest in its involvement in cerebrovascular diseases, particularly ischemic stroke [9]. In the present study, patients with acute ischemic stroke demonstrated elevated SUA levels, and higher SUA concentrations were associated with greater functional disability as assessed by the mRS at presentation.

The demographic profile of the study population showed a predominance of older adults, with a mean age of 59.40±14.5 years, and a higher proportion of male patients. Hypertension and diabetes mellitus were the most frequently observed comorbidities. These findings are consistent with previously published literature. Kante reported that most stroke patients were between 61 and 70 years of age, with a male-to-female ratio of 1.7:1 and a high prevalence of hypertension, diabetes, cardiovascular disease, smoking, and alcohol consumption among hyperuricemic individuals [17]. Ashaduzzaman et al. observed that the majority of patients belonged to the 51- to 70-year age group, again with male predominance [18]. Chinnammanavar et al. also reported a higher incidence of stroke among males (45, 69.23%), with hypertension, diabetes, and coronary artery disease being common associated conditions [19].

In the current study, most biochemical and metabolic parameters were within normal limits; however, the mean SUA level (8.0±1.5 mg/dL) was above the normal reference range, indicating mild hyperuricemia among patients with acute ischemic stroke. This observation is in agreement with earlier studies reporting elevated SUA levels in stroke patients. Sekhar et al. showed that increased SUA levels were significantly associated with age, hypertension, diabetes, dyslipidemia, and coronary artery disease, suggesting that hyperuricemia frequently coexists with established vascular risk factors [20]. However, they did not find a direct association between SUA and cerebrovascular accidents. Similarly, Chinnammanavar et al. did not observe a significant relationship between SUA levels and stroke severity as assessed by NIHSS or mRS, except for an association with low serum calcium levels, highlighting variability in findings across different populations [19].

Patients with higher mRS scores had correspondingly higher mean SUA concentrations, and this association was statistically significant, indicating that increased SUA levels were associated with greater functional impairment following acute ischemic stroke rather than serving as an independent severity determinant. These findings are consistent with those reported by Kante et al., who demonstrated a strong correlation between SUA levels and stroke severity (Spearman’s r=+0.6979), with significantly higher SUA concentrations among hypertensive and diabetic patients [17]. Tutar et al. also reported significantly higher uric acid levels in stroke patients compared to controls (5.5 vs. 4.8 mg/dL; p<0.0019), further supporting the association between hyperuricemia and ischemic stroke [21]. Ashaduzzaman et al. similarly observed elevated SUA levels among stroke patients compared to non-stroke individuals [18].

Additional evidence supporting the potential prognostic relevance of SUA has been provided by Yogiswaran et al., who reported significantly higher SUA levels in stroke patients (6.94±1.97 mg/dL) compared to controls (5.49±1.27 mg/dL), even after adjustment for traditional vascular risk factors [22]. Khanna et al. identified specific SUA cut-off values associated with severe stroke and mortality, reporting that SUA levels ≥7.35 mg/dL predicted severe stroke based on NIHSS scores, while levels ≥5.95 mg/dL were associated with poor functional outcomes and mortality as assessed by mRS [23]. However, these findings should be interpreted cautiously, as SUA levels are influenced by multiple metabolic and vascular factors that commonly coexist in stroke patients.

Strengths and limitations

The strengths of this study include early measurement of SUA levels within 48 hours of stroke onset, use of a standardized and validated functional outcome measure (mRS), and inclusion of radiologically confirmed cases of acute ischemic stroke. However, the cross-sectional design limits causal inference, and the relatively small sample size from a single center may restrict the generalizability of the findings. The use of mRS at admission as a surrogate marker of stroke severity, the absence of National Institutes of Health Stroke Scale (NIHSS) scoring, lack of multivariate adjustment for potential confounders, and unequal group sizes across mRS categories may have influenced the observed associations. Long-term functional outcomes, mortality, and pre-stroke serum uric acid levels were not assessed, and residual confounding cannot be excluded. Larger, prospective, multicentric studies are required to further establish the prognostic utility of serum uric acid in acute ischemic stroke.

## Conclusions

The present study demonstrates that elevated SUA levels are associated with greater functional disability in patients with acute ischemic stroke, as assessed by mRS at presentation. A progressive increase in SUA concentrations was observed with increasing mRS scores, showing a positive correlation. These findings indicate an association between SUA levels and functional disability rather than a causal or independent prognostic role. SUA may represent a simple and readily available laboratory parameter that correlates with stroke-related disability in the acute setting; however, larger prospective studies with multivariable adjustment are required to further clarify its clinical and prognostic significance.
